# The effect of polyethylene cover intervention on ocular surface disorder of intensive care unit patients: a systematic review and meta-analysis

**DOI:** 10.1186/s12886-024-03360-6

**Published:** 2024-03-06

**Authors:** Maryam Askaryzadeh Mahani, Monirosadat Nematollahi, Fatameh Bahramnezhad, Jamileh Farokhzadian

**Affiliations:** 1 Department of Nursing, School of Nursing and Midwifery, Bam University of Medical Sciences, Bam, Iran; 2 Reproductive Health, Family and Population Research Center, Kerman University of Medical Sciences, Kerman, Iran; 3 Department of Critical Care Nursing, School of Nursing and Midwifery, Tehran University of Medical Sciences, Tehran, Iran; 4 Nursing Research Center, Kerman University of Medical Sciences, Kerman, Iran

**Keywords:** Polyethylene covers, Ocular surface disorders, OSD, Intensive care unit, ICU

## Abstract

**Background:**

Polyethylene covers have been proven to be effective in protecting the eyes in patients with decreased or disappeared blink reflexes, but their advantages compared to other conventional methods are still unclear. This systematic review and meta-analysis study aimed to elucidate the impact of polyethylene covers in the prevention of ocular surface disease (OSD) in patients admitted to the intensive care unit (ICU).

**Methods:**

We searched the Scopus, PubMed, Web of Science, and Cochrane Central Register of Controlled Trials (CENTRAL) databases to identify randomized controlled trial studies. This study followed the PRISMA guidelines and used the Cochrane Collaboration tool to assess the risk of bias.

**Results:**

The findings were expressed as risk ratio (RR) with 95% confidence intervals. The incidence of OSD in the polyethylene cover group was lower than that in the eye drops group (RR = 0.27; 95% CI (0.07, 1.09), *P* = 0.07) and adhesive tape group (RR = 0.11, 95%CI (0.04, 0.31), *P* < 0:0001) but the polyethylene cover group showed no significant difference compared to the eye gel group (RR = 0.79, 95%CI (0.18, 3.51), *P* = 0.76) and the eye ointment group (RR = 0.85; 95% CI (0.36, 1.99), *P* = 0.71).

**Conclusion:**

This study showed that polyethylene covers, eye gels, and eye ointments had an equal effect on preventing OSD in ICU patients, and eye drops and adhesive tapes were relatively less effective. However, other intervention methods had not been compared due to the small number of articles. Hence, further studies should assess the available methods to choose the best practical method.

## Background

The ocular surface system includes the cornea, conjunctiva, eyelids, and lacrimal glands. Ocular surface disease (OSD) indicates damage to these surface layers [1]. Healthy eyes have several protective mechanisms, including blink reflex, adequate tear production with antimicrobial activity, and the Bell’s phenomenon- the movement of the eyeballs in an upward and outward direction when the eyelids are forcefully closed [2]. Tears have antimicrobial properties that destroy microorganisms and moisturize the eye surface [3].

Eye injuries in Iran can vary from mild infections to severe damage such as corneal rupture and can even cause permanent damage leading to the loss of vision [4, 5]. Although the prevalence of ocular surface disease (OSD) in ICU patients is very high, the symptoms in most cases are not diagnosed or treated [6], and affected patients receive insufficient clinical attention [7].

Many eye complications are associated with prolonged admission to the ICU (2.8 times in patients admitted to ICU for more than seven days), depressed level of consciousness (seven times), prolonged tracheostomy, positive end-expiratory pressure (2.9 times), and medications (4.2 times in patients receiving sedatives and 2.3 times in patients receiving muscle relaxants) [1]. These complications can cause a spread of infection, perforated cornea, and loss of sight [8]. Therefore, the risk of eye complications increases in ICU patients with a depressed level of consciousness due to impaired eye protective mechanisms, such as reduced tear production and blink reflex [9]. Nursing diagnosis in the North American Nursing Diagnosis Association (NANDA) has focused on eye complications or damage to the cornea and conjunctiva caused by deficiencies in the tear-producing layer [10] and the nursing interventions classification (NIC) has highlighted eye care to prevent irreversible injuries in hospitalized patients [11].

Eye care is one of the most effective yet simple forms of care required in the care of ICU patients, and it is known as the basic care to prevent eye complications [9]. While 80% of vision disorders can be prevented or treated [10], prevention is cheaper, more practical, and more beneficial than treatment [12]. Nurses must start early diagnosis and prevent eye disorders in critical care patients (10). It would be an unimaginable tragedy if patients recover from a life-threatening illness but wake up with visual impairments [13].

Polyethylene covers, eye drops, eye gels, and eye ointments are the most frequent treatments to clinically prevent corneal damage [14]. In Iran, washing eye secretions with sterile normal saline is considered part of routine eye care for ICU patients, but this procedure is not empirically well documented and is not recommended worldwide [8]. Despite sporadic intervention studies in Iran, probably because of the lack of proper and appropriate tools, eye care is not adequately performed. Thus, the rate of eye problems, including eye infections, is still high [15].

Unfortunately, healthcare professionals in different countries use different criteria when selecting eye care interventions in ICUs [3, 8]. Various studies have warned that eye care regimens are not always safe and there is no adequate evidence for the best eye care interventions [9, 16, 17].

Some studies have demonstrated that polyethylene covers protect against corneal ulcers more than eye moisturizers [1, 18], but other studies found a statistically significant difference between the use of polyethylene sheets and moisturizers, with eye closing not being related to chemosis [19, 20]. This systematic review and meta-analysis aimed to examine the impact of polyethylene covers on the prevention of OSD in patients of all age groups admitted to the ICU to provide deeper insights for both patients and healthcare providers.

## Method

### Protocol and registration

We systematically reviewed studies assessing eye nursing care interventions using the Preferred Reporting Items for Systematic Reviews and Meta-Analysis (PRISMA) [21]. The protocol for this study was registered with ID CRD42021288586 in the International Prospective Register of Systematic Reviews (PROSPERO).

### Search strategy

Using keywords and possible Mesh-based combinations, we systematically searched electronic databases of Medline, Web of Science, Scopus, and Cochrane Central Register of Controlled Trials (CENTRAL), as well as Persian databases (SID and Magiran) to find articles published in Persian and English from January 2022 to April 2022. We included all available scientific articles regardless of their date of publication. Two researchers independently searched for Persian and English keywords using the Boolean operator “and/or”: (1) polyethylene cover, (2) Ocular surface disorders or OSD, (3) critically ill patients, (4) intensive care unit or ICU, and (5) critically eye care. All these words were combined (The following search strategy was used: (polyethylene cover OR polyethylene cover*) AND (critical eye care OR intensive care unit OR ICU) AND (“clinical trial” OR “randomized controlled trial” OR “controlled clinical trial” OR RCT). The reference lists of selected studies and systematic reviews were manually searched. Additional papers were obtained from published reviews and reference lists of papers. Disagreements between the reviewers were resolved by consulting a third author.

### Inclusion and exclusion criteria

**Inclusion**: The eligibility criteria were based on the PICOTS question: P (participant: patients admitted to the ICU), I (intervention: polyethylene covers delivered through nursing care), C (comparison: other chemical drugs, herbal medicine, or routine eye care), O (Outcome: efficacy of polyethylene covers on OSD), T (time: length of stay in the ICU), S (study design: randomized controlled trials). The articles were selected with no restriction regarding patient age, country, race, gender, publication language, and date.

#### Exclusion

Studies on eye polyethylene covers in non-human subjects, studies with unreliable data, repetitive or overlapping data, abstract-only papers as preceding papers, conference, editorial, and author responses, theses and books, articles without available full texts, case reports, case series, and systematic review studies.

### Quality assessment of articles

The quality of the studies was assessed by two authors independently based on Cochrane’s guidelines [22]: (a) ‘random sequence generation (selection bias); (b) ‘allocation concealment (selection bias); (c) ‘blinding of participants and personnel (performance bias); (d) ‘blinding of outcome assessment’ (detection bias); (e) incomplete outcome data (attrition bias); (f) ‘selective reporting (reporting bias); and (g) ‘other bias’. In this assessment, each item was marked with a ‘low, ‘high,’ or ‘unclear’ risk of bias [23].

### Study selection and data extraction

**Fig. 1:**
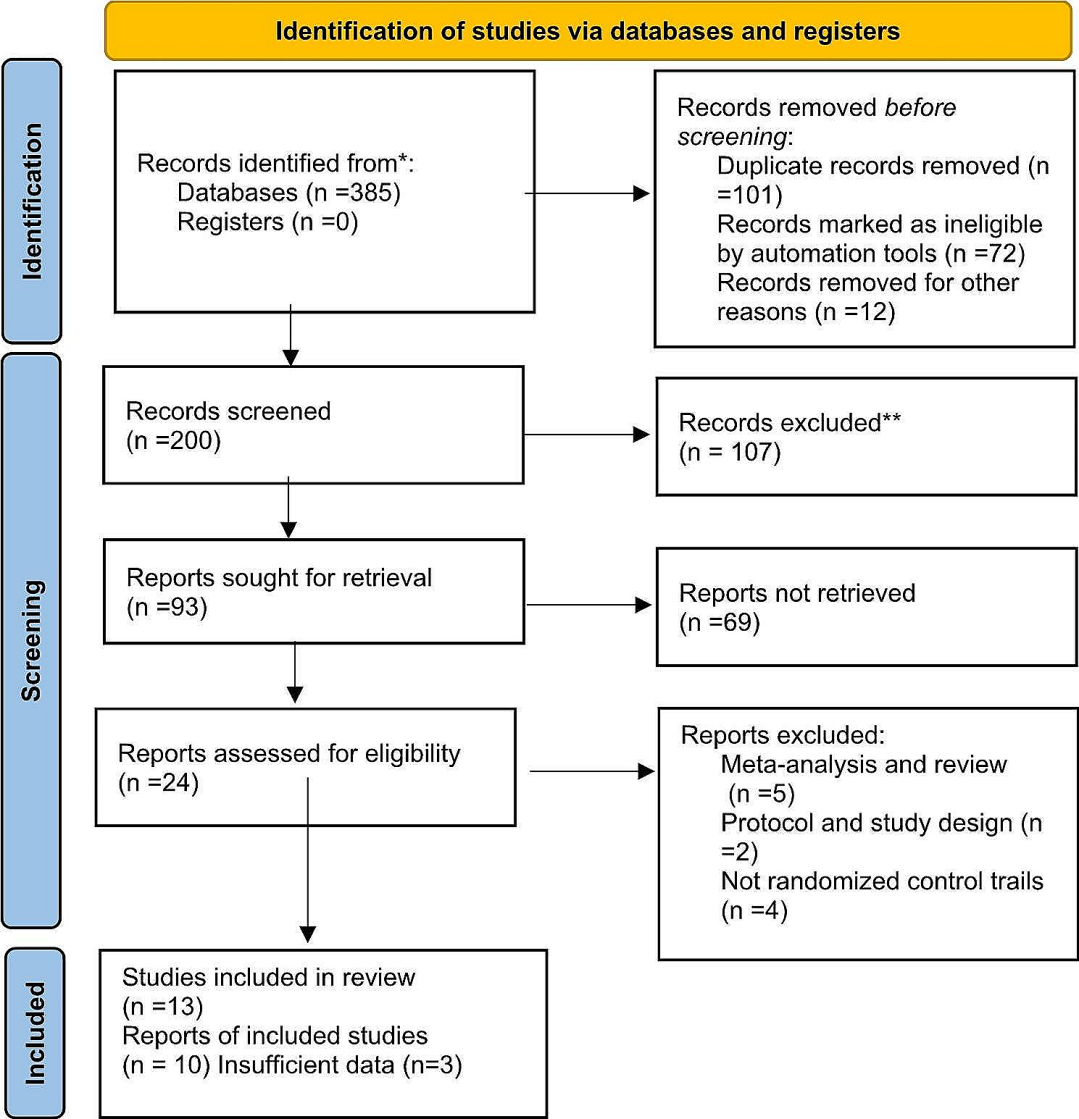
PRISMA 2020 flow diagram. *From*: Page MJ, McKenzie JE, Bossuyt PM, Boutron I, Hoffmann TC, Mulrow CD, et al. The PRISMA 2020 statement: an updated guideline for reporting systematic reviews. BMJ 2021;372: n71. Doi: 10.1136/bmj.n71

In the process of collecting data, 385 articles were assessed by the researchers, and those related to the research objectives were extracted and recorded. All references were included in EndNote software after the search. After removing 185 repetitive articles, 200 remaining articles were reviewed, and the articles related to this study were selected. Initially, the retrieved articles were assessed in terms of relevance, and 107 unrelated articles were removed. In the next step, all 93 remaining articles were examined for access to their full texts and 69 articles whose texts were not related to the purpose of the study were removed. Moreover, 24 articles (5 meta-analyses and systematic reviews, 2 protocols, 4 non-randomized control trials, and 3 insufficient data) were removed. Thus, only ten articles that met the inclusion criteria were selected (Fig. 1). Finally, different items were extracted from each study, including authors, year, country, sample characteristics, and methodological quality (Table 1). The summary tables were thoroughly assessed by three reviewers independently, with a critical discussion of the extracted data.

### Analysis

We extracted and grouped the findings based on the research questions addressed in 13 studies. The main findings that indicated the effect of using polyethylene covers on OSD of patients admitted to the ICU were noted as evidence. Thirteen articles were retrieved from 2004 to 2021, including four from Iran, two from China, one from Australia, one from Turkey, one from Chicago, and one from Saudi Arabia. The articles were randomized clinical trials (RCT).

## Results

### The effect of polyethylene covers on corneal abrasion

The cornea is an avascular layer composed of stratified, non-keratinized, and non-secretory epithelium. It relies on the tear film to maintain adequate corneal wetting and carry oxygen particles during aerobic metabolism. Corneal abrasion (also known as the scratched eye or scratched cornea) is an eye injury that causes significant discomfort, photophobia, and erythema.

Four studies assessed the effect of polyethylene covers on corneal abrasion: The first one suggested that polyethylene covers were as effective as the Hypromellose/lace-lube combination (HL) in reducing the incidence of corneal damage in ICU patients. This randomized controlled trial study found no statistically significant difference between HL and polyethylene [24]. The second study reported that a polyethylene cover was effective in preventing corneal abrasions when compared with lanolin eye ointments, but found no statistical difference in the prevention of keratitis between polyethylene covers and lanolin eye ointments in ICU patients. The incidence of corneal abrasions was not statistically significant (*P =* 0.519) [25]. The third study indicated that polyethylene covers and Viscotears gels were equally effective in preventing corneal abrasions in critically ill patients (*P =* 1.000) [26]. The fourth study observed no difference in the incidence of corneal abrasion between patients undergoing eye cleansing and lubrication with or without the use of a moisture chamber [27].

These four studies concluded that the effect of polyethylene covers was the same as HL, lanolin eye ointments, and Viscotear gels in the treatment of corneal abrasion and found no statistically significant difference in the incidence of corneal abrasions between the control and intervention groups.

### The effect of polyethylene covers on lagophthalmos

In this review, only one study investigated the effect of polyethylene covers on lagophthalmos and suggested that the use of polyethylene covers had no significant effect on lagophthalmos compared with routine eye care in the control group [14].

### The effect of polyethylene covers on dry eye

Two studies assessed the effect of polyethylene covers on dry eyes. The first study suggested that polyethylene covers were significantly effective in preventing dry eye syndrome in ICU patients [12] while the second study showed that both polyethylene covering and polyethylene covering with artificial tear drops were more effective than the conventional method, but polyethylene covering with artificial tear drops was clinically more effective. Therefore, its use is recommended in critically ill patients [28].

### The effect of polyethylene covers on exposure keratopathy

Three studies assessed the effect of polyethylene cover on exposure to keratopathy. The first study recommended that polyethylene covers were more effective and time-saving in reducing the incidence of corneal damage in ICU patients [29]. The second study reported that polyethylene covers were significantly more effective in preventing keratopathy than other methods (*P* = 0.001) and suggested it as a non-aggressive and non-pharmaceutical nursing and therapeutic method for the prevention of keratopathy in patients admitted to the ICU [8]. The third study indicated that carbomer eye drops were effective in managing exposure keratopathy when used in combination with polyethylene covers (*P* = 0.001) [15].

### The effect of polyethylene covers on OSD

Three studies assessed the effect of polyethylene covers on OSD. The first study reported that ointments along with a polyethylene cover as a moisture chamber were considerably effective in preventing keratopathy. Although the odds of OSD in the polyethylene cover group were lower than that of the ointment group, no significant difference was found between the groups (*P* = 0.08) [30]. The second study demonstrated that polyethylene covers were the best intervention for reducing the incidence and severity of OSD in comatose patients [8]. The third study showed that vitamin A eye ointment was more effective than polyethylene covers in preventing OSD in ICU patients [31]. Two of these studies suggested that the use of polyethylene covers was the best intervention in reducing the incidence of OSD in ICU patients.

### Meta-analysis findings

For the meta-analyses, to manage studies with insufficient data, the researcher e-mailed or contacted the corresponding authors to obtain more information so that she could calculate the risk ratio for the selected studies. Then, the studies were separated into subgroups to reduce heterogeneity and improve the interpretation of the findings. Therefore, the studies were divided in terms of the interventions used: (a) eye drops [7, 12, 24, 28, 29]; (b) eye ointments [25, 27, 30, 31]; (c) eye tapes [29, 30]; and (d) eye gel [24, 26].

The reason for repeating the names of the authors is that these studies compared the effect of polyethylene with two or more other interventions. This meta-analysis included 10 RCTs that involved 959 patients, of whom 498 received polyethylene cover treatment and 461 received control treatment (drops, gels, ointments, or eye tapes).

### Statistical analysis

Review Manager (RevMan version 5.4; Cochrane Collaboration) was used for the meta-analysis. The chi-squared test and I^2^ were used to detect heterogeneity among the studies. The *I*^*2*^ values exceeded 25%, 50%, and 70%, indicating low, moderate, and high heterogeneity among the studies. It is generally believed that an I^2^ ≥ 50% indicates substantial heterogeneity. The fixed-effects model was used to analyze homogeneous studies; otherwise, a random-effects model was used to calculate the pooled findings. Dichotomous outcomes (OSD and NO) were represented as risk ratios (RR) and pooled using inverse variance weighting. RR with a 95% confidence interval (CI) was used to estimate the effectiveness. A sensitivity analysis was performed by excluding the studies one by one.

#### Publication Bias

The visual inspection of the funnel plot asymmetry was used to assess publication bias.The methodological quality of the included RCTs ranged from moderate to high. The sample sizes of the included RCTs ranged from 18 to 207 patients. Some researchers performed a meta-epidemiological study and indicated that small to moderately-sized trials had stronger effect estimates than larger trials [32]. Therefore, we used a comparison-adjusted funnel plot to assess the bias of small study effects. The funnel plot was visually checked and the shape was relatively asymmetric (Fig. 8). The findings indicated a low probability of bias in the included studies.

### Characteristics of the studies

Thirteen articles (76.9%) were published in the last 10 years (2010–2021), and 30.7% (*n* = 4) of the studies were multicenter. Most of the articles were from Asia (61.5%; *n* = 8). The fluorescein staining test was the most commonly used method to measure the effectiveness of the interventions (100%) (Table 1).

**Table 1:** Description of the studies included in the systematic review (*n* = 13)

Authors (year)	Country	Aim	Design	Comparison (Control) group	Sample and study population	Data collection	Method of analysis
Kocaçal 2021 [16]	Turkey	To compare the effect of carbomer eye drops when used alone and in combination with polyethylene covers in the healing of exposure keratopathy	RCT	The control group received only carbomer, eye drops while the intervention group received both carbomer eye drops and polyethylene covers	24 patients	fluorescein stain test	Chi-square test, Friedman Test, Wilcoxon Signed-Rank Test, and Mann-Whitney U Test
SO, 2008 [25]	China	Comparing the effectiveness of polyethylene covers (GladwrapTM) with lanolin (Duratears1) eye ointment in the prevention of corneal abrasions in critically ill patients	RCT	The control group received lanolin eye ointment while the intervention group received polyethylene covers	116 patients	fluorescein stain test	Fisher’s exact test, Kaplan-Meier analysis
Güler 2010 [3]	Turkey	Comparing polyethylene covers and carbomer eye drops for critically ill patients	RCT	One eye of the patient was randomly covered with a polyethylene cover every 12 h, and carbomer drops were instilled in the other eye every six hours	18 patients	Schirmer and fluorescein tests	The Wilcoxon signed-rank test Mann–Whitney U test Chi-square test, Spearman Correlation
Sorce 2009 [27]	Chicago	To determine whether a moisture chamber over the eye is more effective in preventing corneal abrasions compared with standard therapy	RCT	The experimental group received the instillation of a ribbon of lubricating petrolatum white and ophthalmologic ointment and maintenance of a small piece of plastic film over the eye. The control received the standard of care in the units	207 Patients	fluorescein stain	Fisher’s exact test, Wilcoxon’s, and McNemar’s test
Ahmadinejad 2020 [30]	Iran	To compare three eye care methods to prevent OSD In ICU patients	RCT	Three groups using the six-person blocks. In the first block, 2 patients received polyethylene cover and eyelid taping; in the second block, 2 patients received polyethylene cover and simple eye ointment; and in the third block, 2 patients received eyelid taping and simple eye ointment	152 Patient	slit lamp with fluorescein staining	ANOVA and chi-square tests Logistic regression and zero-inflated Poisson regression
Koroloff 2004 [24]	Australia	compare the efficacy of two forms of eye care (hypromellose and Lacri-Lube combination vs. polyethylene/Cling wrap covers) for ICU patients	RCT	group one (*n* = 60) received a treatment combining hypromellose drops and LacriLube (HL) Group two (*n* = 50) had polyethylene covers only placed over the eye	110 patients	fluorescein stains and mobile slit lamp	Chi-square. T- Test, Mann-Whitney U, and Fisher’s exact tests
Khatiban 2021 [7]	Iran	To compare the use of polyethylene eye covers and artificial teardrops versus normal saline on the incidence and severity of OSD in comatose patients	RCT	group A, patients received artificial teardrops for left and normal saline for right eyes, in group B polyethylene covers for left and normal saline for right eyes, and in group C polyethylene covers for left and artificial teardrops for right eyes	90 patients	Fluorescein Staining Pattern	Chi-Square One-way analysis of variance (ANOVA) McNemar’s test the Kruskal–Wallis’s test
Kalhori 2016 [8]	Iran	compare the effect of three eye care techniques in the prevention of keratopathy	RCT	polyethylene cover, liposic ointment, and artificial tear drop randomly on one eye of each sample and a comparison was made with the opposite eye as the control	96 patients	Fluorescein Staining and portable slit lamp	chi-squared test, Fisher’s exact test, ANOVA, and Kruskal–Wallis one-way analysis of variance
Nikseresht 2019 [28]	Iran	To compare the effectiveness of polyethylene cover versus polyethylene cover with artificial tear drop to prevent dry eye in critically ill patients	RCT	one eye was randomly selected as a control eye and the other was considered as an intervention eye	104 patients	Schirmer and fluorescein tests	Kolmogorov–Smirnov test, independent t-test, Friedman test, Fischer’s exact test and Mann–Whitney U-test)
Shan 2010 [29]	China	To compare the efficacy for preventing exposure keratopathy of three forms of eye care (artificial tear, moist chamber, and polyethylene covers) for ICU patients	RCT	Three treatment groups, including artificial tears group, moist chambers group, and polyethylene film group	84 patients	fluorescein stains	One-way analysis of variance, Tukey’s tests, and Chi-square test
Babamohamadi 2018 [31]	Iran	determine the effectiveness of vitamin A eye ointment (VAEO) and moist chamber (MC) in preventing OSD in ICU patients	RCT	patients were randomly administered VAEO in one eye every and a polyethylene cover (PC) was placed on their other eye	38 patients	Fluorescein stain examination and Schirmer’s tests	Wilcoxon, McNemar, and Spearman tests
Al-Ribh 2012 [26]	Saudi Arabia	Comparing the effectiveness of polyethylene covers with Viscotears gel in the prevention of corneal abrasions in critically ill patients	RCT	All participants randomly receive polyethylene covers in one eye and viscotears gel in the other eye	40 patients	fluorescein stain test	Mann-Whitney test and Fisher’s exact tests, McNemar’s test
Nikseresht 2021 [14]	Iran	Comparing the Effect of Three Eye Care Methods on the Severity of Lagophthalmos in ICU Patients	RCT	The control eye received routine interventions in one eye. Experimental group “polyethylene cover,” polyethylene cover plus artificial tear drops,” and “polyethylene cover plus Lubratex eye ointment.” In another eye	156 patients	lagophthalmos intensity checklist, fluorescein stain strips	ANOVA, Friedman test, Wilcoxon test, Kruskal–Wallis test

### Quality assessment

**Fig. 2:**
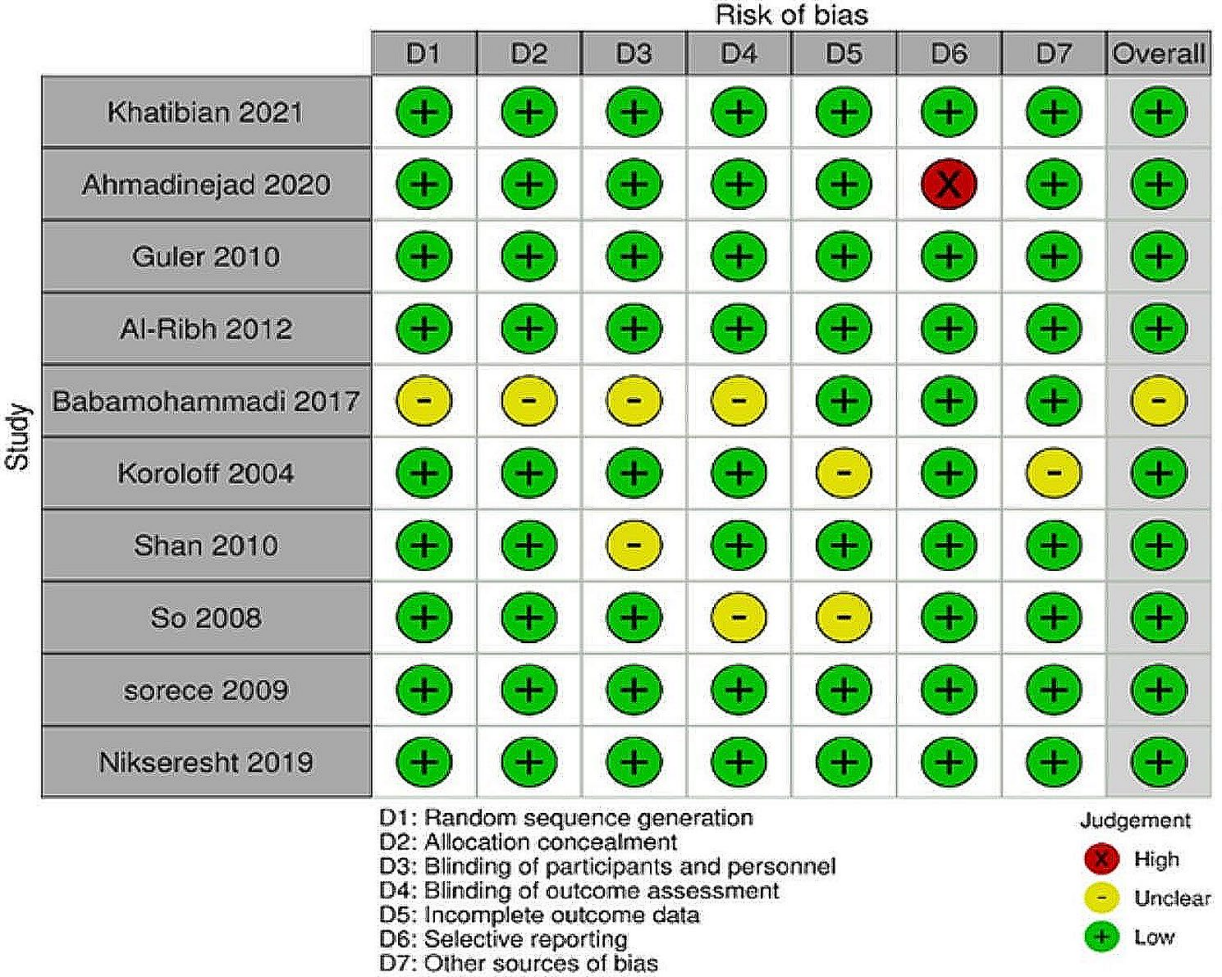
Risk of bias summary in the included studies

**Fig. 3:**
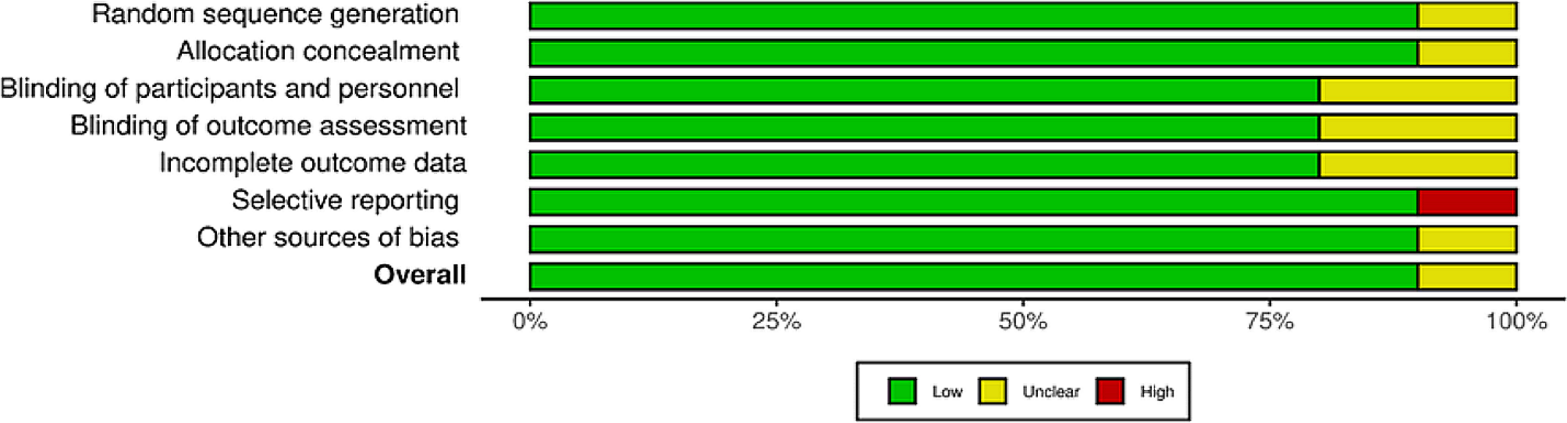
Quality assessment of the included studies: low risk (green color), unclear (yellow color), and high risk (red color)

A bias risk was assessed at the study level, and methodological quality was assessed using the Cochrane bias risk assessment tool. After reading the full texts, the author found a high risk of reporting bias in one study (Fig. 2). Figure 3 summarizes the risk of bias in each study.

### Heterogeneity test

#### Polyethylene covers versus eye drops

**Fig. 4:**
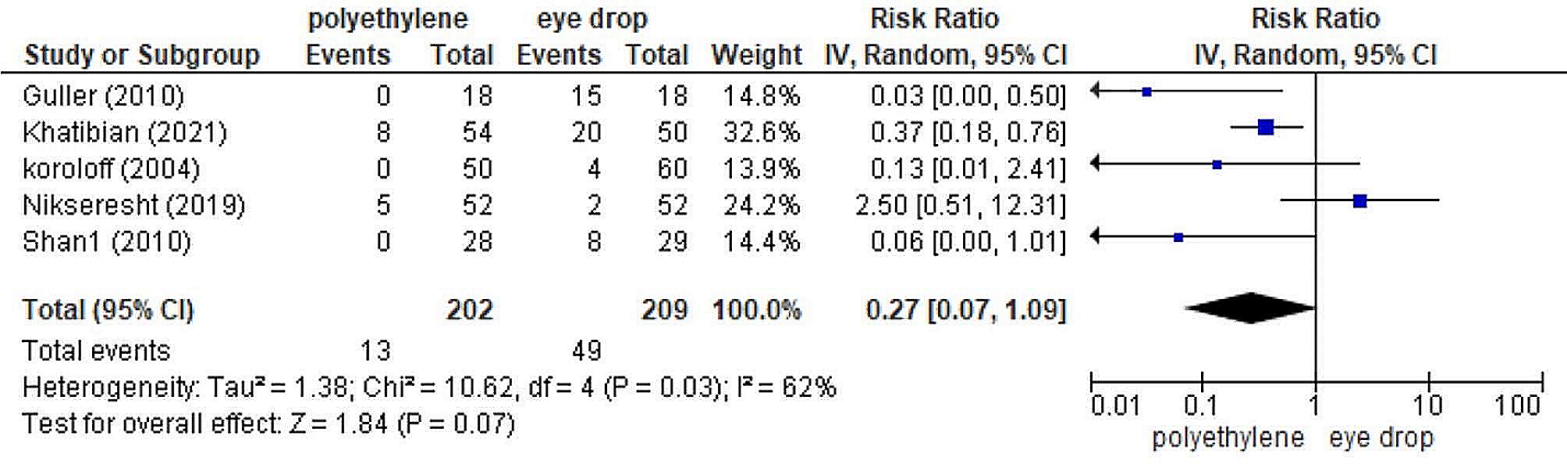
Forest plot: comparison of incidence of OSD between polyethylene cover group and eye drops group. RR: risk ratio

The Q Cochran test demonstrated the heterogeneity between the findings of the Polyethylene cover group and eye drops group, and thus a random model of the meta-analysis was used instead of a fixed model (*RR =* 0.27; *95% CI (*0.07, 1.09*)* (Fig. 4). The findings showed that polyethylene covers were significantly more effective than eye drops in preventing OSD in ICU patients (*P =* 0.07) [8, 12, 24, 28, 29].

The values of the heterogeneity index (*P =* 0.03; *I*^*2*^ = 62%) confirmed moderate heterogeneity between the studies. Sensitivity analysis was performed to detect potential resources in our meta-analysis. Nikseresht et al.’s study [16] had potential resource heterogeneity and the removal of this study decreased heterogeneity to 31% (*RR =* 0.17; *95% CI*: 0.05, 0.57; *p* = 0.23; *I*^*2*^ *=* 31%).

### Polyethylene covers versus eye ointments

**Fig. 5:**
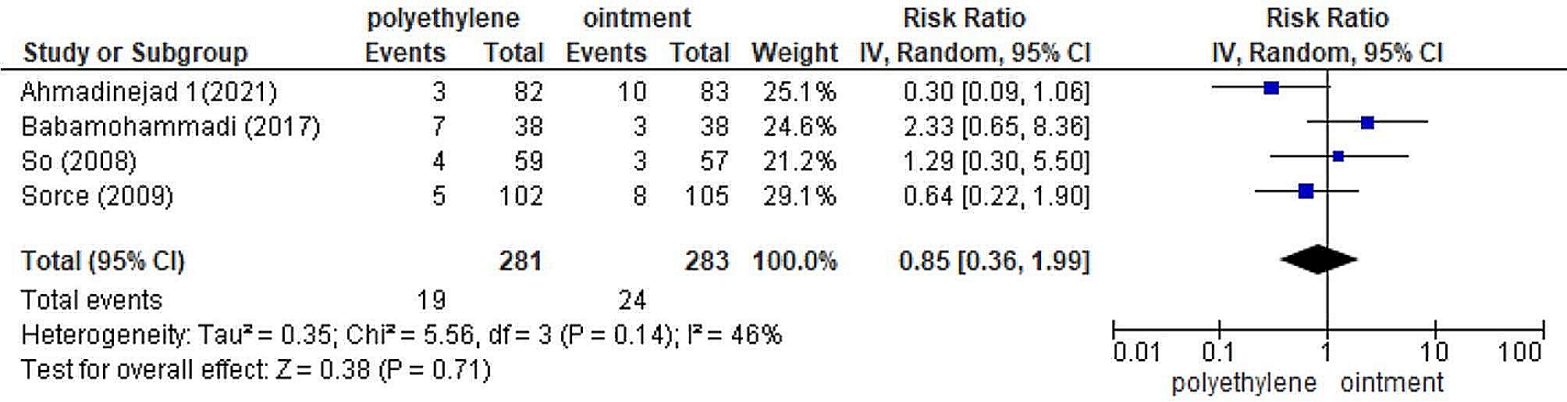
Forest plot to compare the incidence of OSD between the polyethylene cover group and eye ointment group. RR: risk ratio

The analysis showed no statistically significant difference between polyethylene covers and eye ointments on the incidence of OSD in ICU patients *(P =* 0.71*)* [25, 27, 30, 31] (Fig. 5). The heterogeneity between the studies was moderate *(RR =* 0.85; *95% CI*: 0.36, 1.99; *P =* 0.14; *I*^*2*^ *=* 46%*)*. A sensitivity analysis was performed to exclude Baba Mohammadi et al.’s study [31]. The heterogeneity changed after the removal of the mentioned study (*RR =* 0.60; *95% CI*: 0.28, 1.27; heterogeneity; *P =* 0.33; *I*^*2*^ *=* 10%; random effect model).

### Polyethylene covers versus adhesive tape

**Fig. 6:**
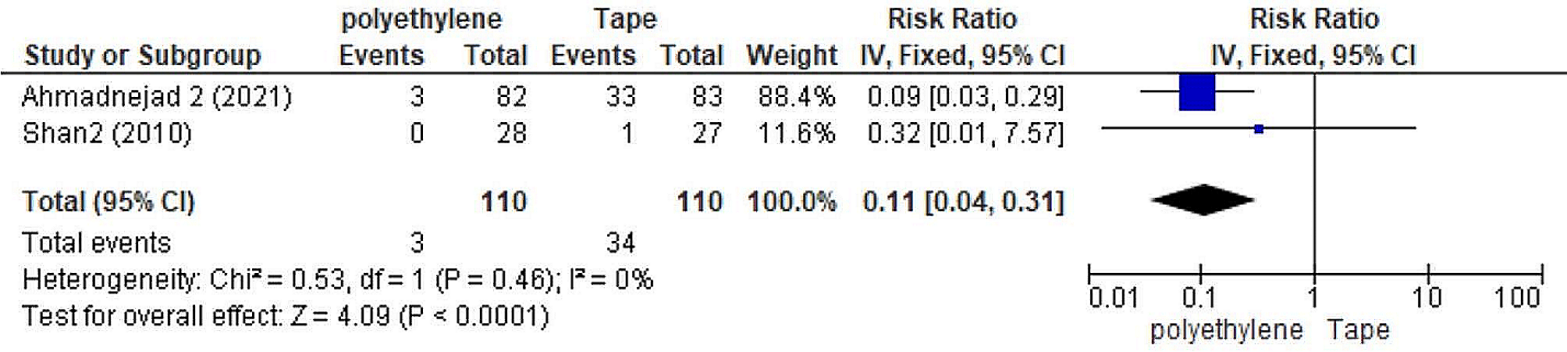
Forest plot to compare the incidence of OSD between polyethylene cover group and eye adhesive tape group. RR: risk ratio

The analysis of polyethylene covers in two studies [29, 30] (Fig. 6) showed that ICU patients in the polyethylene cover group had a significantly smaller number of OSD compared to the adhesive tape group *(RR =* 0.11; *95%CI*: 0.04, 0.31; *P =* 0.46; *I*^*2*^ *=* 0%; *P <* 0:0001*)*.

#### Polyethylene covers versus eye gels

**Fig. 7:**
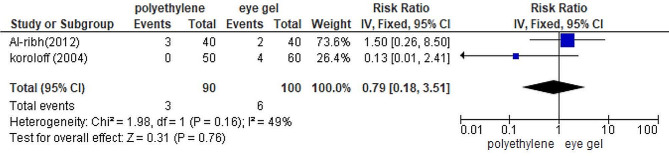
Forest plot to compare the incidence of OSD between the polyethylene cover group and eye gel group. RR: risk ratio

No statistically significant difference was observed between polyethylene covers and eye gel in terms of their effectiveness in preventing corneal abrasions in ICU patients *(P =* 0.76*)* [24, 26] (Fig. 7). *(RR =* 0.79; *95%CI*: 0.18, 3.51; *p =* 0.16; *I*^*2*^ *=* 49%; fixed effect model; 2 trials).

##### Publication Bias

**Fig. 8:**
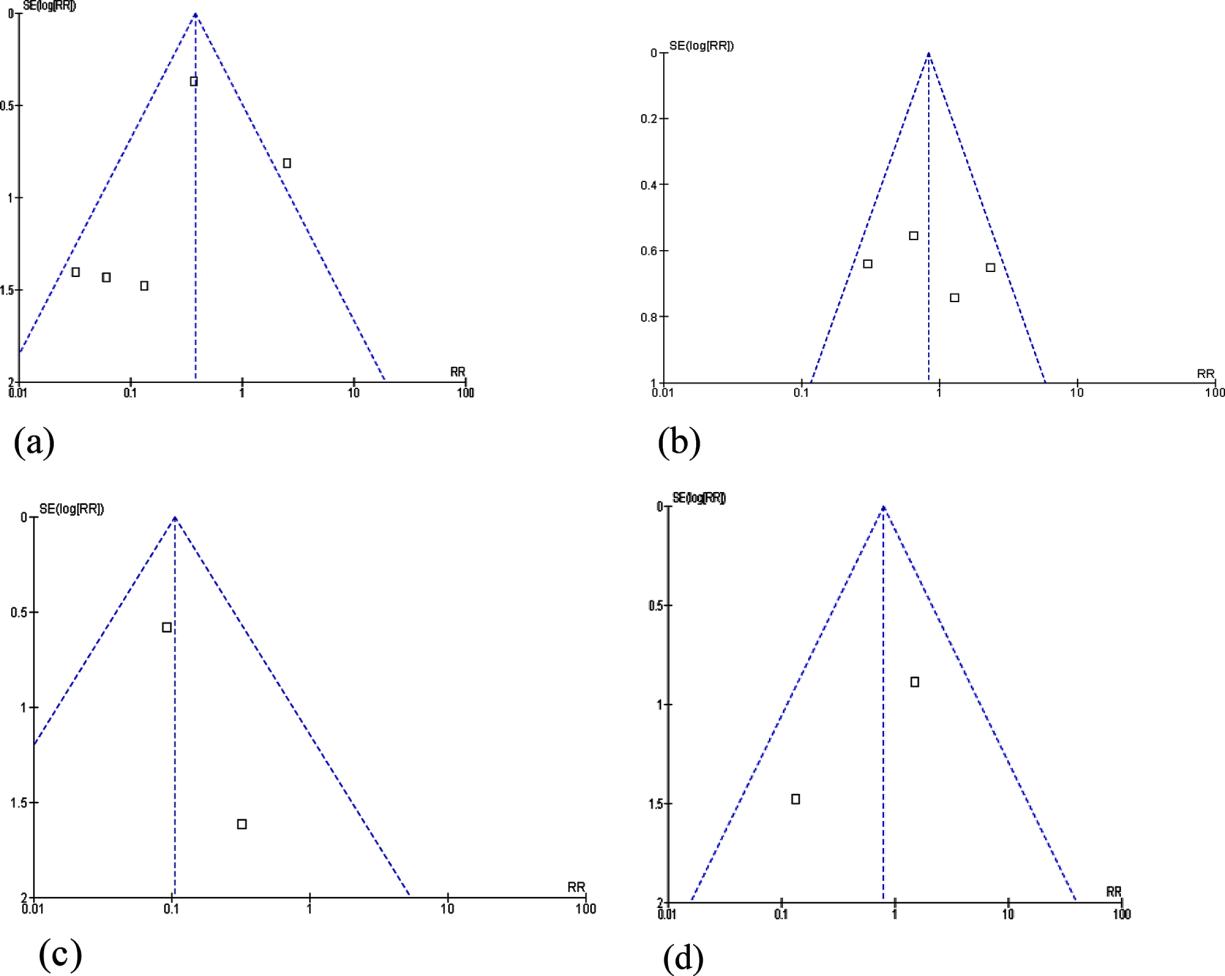
Funnel plot of publication bias: (a) polyethylene cover and eye drop; (b) polyethylene cover and eye ointment; (c) polyethylene cover and eye tape; (d) polyethylene cover and eye gel

The visual inspection of the funnel plot asymmetry was used to assess publication bias (Fig. 8).

## Discussion

The incidence rate of ocular surface disease and other ophthalmic complications in ICU patients is high, ranging from 20 to 60% of cases, depending on the diagnosis criteria, the length of stay at the hospital, and other factors [33]. A very recent study on eye care for ICU patients indicated that lubricants and taping of the lids are only advised for conjunctival and corneal exposure. Polyethylene eye covers were not considered a part of the standard eye care for critically ill patients [8].

Polyethylene covers are made of a single polymer obtained from the polymerization of ethylene, with no potentially toxic substances. A polyethylene cover is approximately 0.01 mm thick, easily adheres to surfaces [12, 16], protects the eyes by creating a moist chamber, prevents the evaporation of tears, keeps the eye surface naturally moist, and prevents microorganisms from entering the eye, especially during suctioning and feeding through a nasogastric tube (NGT) [8]. These easy-to-use covers prevent possible corneal complications and keep the eyes clean and closed [17].

This study presented a meta-analysis of the effect of polyethylene covers on ocular surface disorders in critically ill patients. Ten studies involving 959 critically ill patients were assessed in the meta-analysis. Five studies had compared eye drops [8, 12, 24, 28, 29], four studies had compared eye ointments [25, 27, 30, 31], two studies had compared taping eyes closed [29, 30], and two studies had compared viscotear gels with polyethylene covers [24, 26].

The findings from this study confirmed the effectiveness of polyethylene covers compared to eye drops in preventing ocular surface diseases [6, 17, 18, 34]. However, this finding was contrary to the results reported by Nikseresht (2019) [28] possibility due to the differences in the age and physical conditions of patients, their length of hospital stay, the dose of anesthetics received, and the type of patients admitted to the intensive care unit. Nikseresht et al. studied patients with head trauma and intracranial bleeding who underwent brain surgery and required regular monitoring for pupillary reflex, which disturbed the performance of the polyethylene cover because it had to be removed and replaced during the examination [28].

In a randomized controlled trial study by Kokacal et al. (2021), the patients in the control group received only carbomer eye drops, while the patients in the intervention group received both carbomer eye drops and polyethylene covers. They found that carbomer eye drops in combination with polyethylene covers were effective in managing keratopathy [13]. More than 90% of the ophthalmic drugs available on the market are eye drops, which have low ocular bioavailability (< 5%). A large amount of them is lost by nasolacrimal drainage which shortens the medicinal activity and increases eye dryness in patients with lagophthalmos. Thus, frequent doses are necessary [35].

Our findings showed no statistically significant difference in the incidence of ocular surface diseases between the polyethylene cover group and the eye ointment group. Other studies (e.g., Kalhori, 2016; Yao, 2021; Rosenberg, 2008) found that the ocular surface diseases in the polyethylene group were much less frequent than in the eye ointment group [8, 18, 34]. The difference between the findings may be due to the small sample size, the difference in the type of polyethylene covers or eye ointments used, the low level of accuracy, and the low quality of the studies.

The study findings confirmed the effect of polyethylene covers on preventing ocular surface disease compared to taping the eyes closed. Various studies highlighted that lid taping was not always necessary, could bother the relatives, and its repeated removal might damage or irritate the facial skin or eyelids [38], but Yao (2021) did not support these findings [34]. Hearne (2018) recommended horizontal tape for taping the eyelids, but if vertical tapes used, the eye should be kept closed where there is no tape [36].

The data from this meta-analysis indicated no statistically significant difference in the incidence of ocular surface diseases between the polyethylene cover group and the eye gel group. The exact mechanism of action has not been proven yet, but it seems that ophthalmic gels form a viscous and sticky layer on the ocular surface, which subsequently increases the ocular bioavailability of ophthalmic drugs [36] and functions as a polyethylene cover for the eye.

This systematic review and meta-analysis had some strengths: (1) We only chose randomized controlled trials; (2) we did not consider the age range and included all age groups hospitalized in the ICU, (3) we used a combination of keywords and theme searches in all fields to minimize publication bias, and (4) a meta-analysis was conducted to compare the efficacy of different nursing methods in preventing OSD.

### Limitations

The limited number of studies was the first shortcoming of the present study because it was not possible to evaluate the heterogeneity of studies by meta-regression. The second limitation was the low methodological quality in some evaluated studies due to ineffective randomization and double-blinding techniques and the failure to report intention-to-treat analysis. Thus, future studies should adhere to CONSORT guidelines. The third limitation is this systematic review and meta-analysis was that the resources had been published in peer-reviewed journals without any time limitations. Therefore, gray materials, review articles, commentaries, editorials, and articles presented at seminars and conferences were not included in the review. Finally, despite the effort to contact researchers, it was not possible to enter the study data (*n* = 3) into the Rev Man software, and some studies were deleted, leading to missing the relevant data.

## Conclusion

This study showed that polyethylene covers, eye ointments, and gels were equally effective in preventing ocular diseases in patients admitted to intensive care units, while eye drops and taping eyes closed were relatively less effective. Therefore, further controlled clinical trial studies with adequate sample sizes are necessary to clarify the advantages, disadvantages, and possible side effects of each of these methods. Policymakers and curricular developers can take measures so that care providers fully understand the mechanisms of action and functions of different eye disease prevention methods in patients admitted to intensive care units, choose nursing or treatment procedures according to the number of patients admitted to the intensive care unit and the workload of nurses, select the number of personnel working in intensive care units, and implement individual treatment plans for patients to reduce or stop the occurrence of ocular surface diseases in intensive care units. The findings of this study have some implications for developing evidence-based clinical practice guidelines for the eye care of patients admitted to intensive care units.

## Data Availability

The data that support the findings of this study are available on request from the corresponding author. The data are not publicly available due to privacy or ethical restrictions.

## Abbreviations

OSD: Ocular Surface Disorder
ICU: Intensive Care Unite
GCS: Glasgow Coma Scale
